# A Face to Love or Trust

**DOI:** 10.3390/bs13060494

**Published:** 2023-06-12

**Authors:** Christer Johansson, Per Olav Folgerø

**Affiliations:** Department of Linguistic Literary and Aesthetic Studies, University of Bergen, 5007 Bergen, Norway

**Keywords:** attractiveness, trustworthiness, human bias

## Abstract

This article demonstrates the use of a card sorting game that is suitable for field studies. Subjective judgment in face perception is studied by sorting faces based on attractiveness or trustworthiness. Are beautiful people also trustworthy, or does beauty come with a price? Our first hypothesis is that the two conditions like and trust are different. We investigate this using a sorting game, where participants are asked to sort 27 semi-artificial portraits according to how much they like or trust the faces. The faces are in two different conditions: prototypes and individualized prototypes. Our participants were consistent in their judgments. Participants claim to have reacted to small inconsistencies in facial expressions in the trust condition, and we investigate the relation to anatomical features using a model and Correspondence Analysis.

## 1. Introduction

The goal of this article is to investigate the use of a sorting game for assessing reactions to faces. The material originates in research on symmetry in portraits and has been further developed to investigate prototype effects. One central theme is how judgments of attractiveness and trustworthiness are related. 

Our first hypothesis is that participants will react differently to faces in the like and trust condition, following DeBruine [1,2] and suggested by Lembke et al. [3]. We are also interested in other factors, such as the effect of prototypicality (i.e., the average of all male and female participants, as well as the average between male and female participants). The images of the participants were morphed with the image of an average portrait of Jesus. The second hypothesis is that the participants will react differently to female and male versions of the presented images of Jesus. A third tentative hypothesis is that the participants will react more positively to images that contain facial information from themselves (see Section 2). A fourth tentative hypothesis is that participants react more positively to images that contain portraits from their “own” group, rather than an unknown out-group. Our subjects took part in the same classes, were all ethnic Romanian, and some of them had known each other before their exchange stay at our university. Their faces were also objectively similar according to a simple anatomical model of faces (see Section 3.4).

### Differences between Trustworthy and Lustworthy

A study by Jacobsen et al. [4], using f-MRI studies has shown that Brodmann area (BA) 10 is activated during judgment processes including aesthetics, but also judgment tasks more generally, such as moral and social judgments [4]. Since trustworthiness involves moral and social signs, judgments of it will involve such brain areas. For attractiveness/beauty judgments, other areas will be more prominent. The most plausible areas involve the medial orbitofrontal cortex (BA11, confer [5]) and other reward areas, such as those indicated by Vartanian and Goel [6]:

“*Studies have shown that activations in medial orbitofrontal cortex, left anterior frontal cortex, left frontal-temporal junction, nucleus accumbens, right caudate nucleus, and visual cortex are related to viewing faces of varying degrees of attractiveness [7,8,9]. Activation in the aforementioned regions has been attributed to the reward properties of faces.*”

[6] (p. 893).

Interestingly, Tsukiura and Cabeza, in an f-MRI study, demonstrated that activation of the mOFC (medial orbitofrontal cortex, part of the visceromotor network associated with emotional awareness) and the insular cortex are opposite when it comes to beauty and goodness judgments: both a judgment of attractiveness and of goodness activates the mOFC, whereas the insular cortex is activated during low attractiveness ratings as well as ratings of (hypothetical) bad social actions, giving a “beauty-is-good stereotype” [10].

In other experiments, we have observed that it takes significantly longer to judge trustworthiness, compared to a more automatic reaction to attractiveness. Processing time will depend on several processing stages; while a decision for beauty predominantly depends on an external stimulus only, a simple decision of beautiful or not, judgments of trustworthiness will need to rely on more cognitive processing. The external stimulus must be transformed to become internalized and thus comparable to our internal “catalogue” of experiences (confer [11]). It is suggested that dorsolateral prefrontal cortex is primarily involved with *externally* generated information, while frontomedian prefrontal cortex (BA10) is related to evaluation and manipulation of *internally* generated information. This predicts a heavier cognitive load when conversion is needed from external to internal information. 

External information can be a complex task to process (e.g., trustworthy/not trustworthy); hence, additional internal processing is needed. A case in the research literature involves the difference between judgments of beauty and judgments of symmetry. Drawing on Jacobsen et al., Cela-Conde et al. argue that beauty judgments are subject based, as opposed to the objective symmetry judgments. The abstract geometrical patterns used by Jacobsen et al. “might have increased the subjective elements of such judgments, where participants could only base their decision [beautiful or not] on internally generated information” [12] (p. 43). 

Christoff and Gabrieli [11] suggest a three-step processing model, where the frontopolar region (BA10) promotes a third step of

“*executive processing, involving evaluation of information that has been generated at a previous stage of executive processing. Thus, the ventrolateral, dorsolateral, and frontopolar regions can be seen as forming a three-stage hierarchical system within the prefrontal cortex*”

[11] (p. 183).

Self-generated information derives from the external environment but cannot be processed internally before it is converted into a form that the brain can process [13]. Cristoff et al. found

“*involvement of lateral BA10 during the evaluation of self-generated cognitive information, whereas other functional neuroimaging studies have shown that medial BA10 is activated during judgments of self-generated emotional states […]. This suggests that the entire region may be involved in the explicit processing of internally generated information, with lateral BA10 recruited during cognitively oriented tasks and medial BA10 recruited during emotionally oriented tasks. This ability to become aware of and explicitly process internal mental states—cognitive as well as emotional—may epitomize human mental abilities and may contribute to the enhanced complexity of thought, action, and social interactions observed in humans.*”

[13] (p. 1166).

Sofer et al. [14] argue that trustworthiness judgments peak around the “typical face”, whereas attractiveness increases beyond the typical face. Thus, detecting the face typicality plays an important role in face evaluation. It may be more difficult to detect what is a typical face when the face type is unfamiliar, for example, if the face is from a different ethnic group. We should be clear that what is typical depends on the context and our expectations, just like other linguistic concepts such as hot or cold: what is hot in Antarctica can be extremely cold in the tropics. As humans, we are often very attentive to anything that deviates from our expectations. It is interesting that we can often agree on what is hot or cold or typical, even when there is no common absolute scale. It could be that part of what is typical in an experiment emerge or adapts with exposure to the dataset. The positivity bias towards the familiar, typical faces, for both like and trust, would predict a stronger correlation between such judgments when the face is typical. The typical face could be either absolute, relating to each subject’s experience, or more dynamic, relating to what is typical in the task or the dataset being explored.

This may contribute to an explanation of why the most attractive faces in our study (female prototypes) ranked low for trustworthiness, i.e., the closer to an ideal attractive face, and the less close to the average expected face, the harder it is to infer trustworthiness. In our study, individualized faces are made more typical (within the dataset) by morphing them with a common prototype. Reaction times for evaluation of trustworthiness are longer because the processing fluency is reduced, compared to a judgment of beauty. (For more information on processing fluency, confer [15]). Familiarity may also play a role. Sofer et al. [14] claim that

“*Typicality and perceived familiarity are highly correlated [16,17]. Familiarity enhances positive affect toward objects [18], and familiar faces are liked more and judged to be safer than unfamiliar faces [19]. Taken together, these findings suggest that the high level of perceived trustworthiness of the typical face likely arises from the inherent preference for typicality, mediated by familiarity.*”

[14].

As Hannibal Lecter (Anthony Hopkins) tells Clarice Starling (Jodie Foster) in *The Silence of the Lambs*, “We begin by coveting what we see every day”. This supports the idea that, while attractiveness and trustworthiness are correlated, trustworthiness is different, breaking the correlation when the face deviates from the typical face. We may note that the standard for the typical face may change dynamically when we are exposed to a large set of faces to judge, but the ranking is expected to be robust relative to the more typical faces in the dataset. 

A high ranking is at the top of the distribution, which means low numbers, and a low ranking is at the bottom of the distribution, meaning large numbers (see Table 1 and Appendix A). The start of finding out what is attractive may be in familiarity.

## 2. Methods

The participants were Romanian exchange students (8 aged 18–25, and two older than 25) participating in a course on neuroaesthetics. There were 3 male and 7 female students. All students were informed that their participation was voluntary. At the start of the course, all participants had their portraits taken by a professional photographer. 

**Figure 1:**
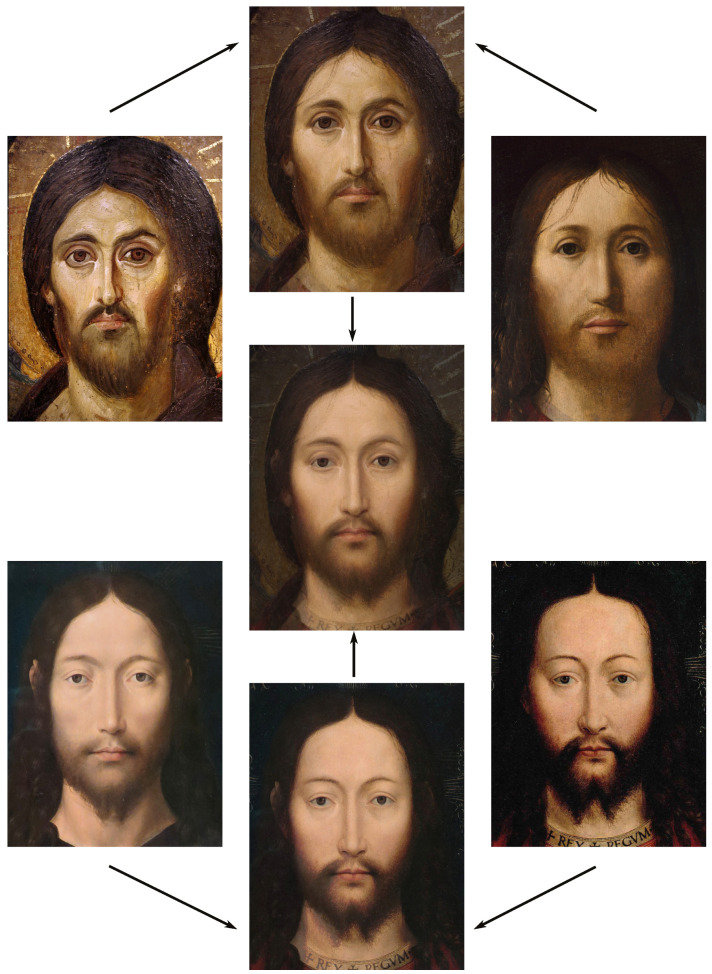
The construction of a prototypical Jesus. The four images, left to right, top to bottom: Christ the Pantocrator, Monastery of Saint Catherine, Sinai, 6th century; Antonello da Messina, Holy Face/Christ blessing, National Gallery, London (apparently dated 1465); Hans Memling, Holy Face, Norton Simon museum, Pasadena (1478); and after Jan van Eyck, Holy Face, Gemäldegalerie, Berlin (1438).

About two to three weeks later, they participated in the study. Part one of the study was a reaction time study and, occurring a week later, part two was a card sorting game. This article will focus on part two. Each portrait was morphed with a prototypical painting of Jesus (80%). The prototypical image of Jesus was constructed by pairwise morphing of four representative images of Jesus (see Figure 1). Our intention was not to create a (prescriptive) norm for what Jesus, or humans, ought to look like, but rather to create a familial fixpoint. The Jesus prototype works as a mask and a constant that is present in the individualized pictures. Different cultures may have different fixpoints, and alternative iconic fixpoints exist. One may even argue that famous people and actors take on iconic qualities that deem them larger than life, for example, Elvis, Prince, Tina Turner, or Ingrid Bergman. Each culture has different iconic images that are larger than life, even though some cultures may avoid them.

The choice of template was motivated by an earlier investigation into preferences in Renaissance paintings and facial symmetry [20]. Another motivation is to be able to focus on more general proportions in the face rather than individual faces. The morphing procedure smooths out differences in skin quality, small blemishes, and minor asymmetries, as noted by Galton [21]. The procedure also serves to hide the identity of the participants, and thus protect the anonymity of the participants outside of the project, and minimizes the risk that the images can be used for the unintended identification of any of our participants. However, we are interested in whether the participants will react differently to their own morphed images, compared to images of others. Familiarity with one’s own face could be assumed, although the face would be most often mirror-imaged.

Images from two earlier studies were also used. All images in this study were constructed in accordance with the procedure described by Lembke et al. [3] and can be viewed in Table 1. Cards were printed and laminated in a 55 × 73 mm format with a thin white border. Each participant was handed an individually shuffled deck of 27 cards and asked to sort the cards according to how well they liked the faces or how well they trusted the faces. Each person was either in the like or trust condition. Liking the face was defined as finding the face more beautiful, or attractive. Trusting the face was exemplified by the question “Would you buy a car from this person?”. Essentially, the instructions differed only on the criteria for sorting; everything else was the same. The cards had to be sorted in five minutes, as measured by an ordinary watch. The experimenter would remind the participants when they had one minute left. Five participants were present in the room for each condition, standing up and placing the cards on tables. Each table was separated by at least one other table. After each session there was a brief debriefing. The main results were presented and discussed in class the week after. The condition in this study was like or trust, and we used image gender, in-or-out group, and self as fixed factors. Significant deviations from the expected (median) ranking were estimated using the chi-square calculation against the expected value (14). This sets the limits at a higher ranking than 6.7 and a lower ranking than 21.3 (i.e., 14 ± sqrt(3.8 × 14)).

One caveat: Two researchers were reading out the card names of the sequences, as one of them entered the data in an Excel spreadsheet. It was later detected that one item was mislabeled, possibly by autocorrect (X10/X), in the trust condition. The score was either 4 or 8. It is impossible to know which of the two items has which score. The average score of 6 was entered for both items for this one participant, affecting two data points.

## 3. Results

There were five participants in each condition, which makes it difficult to find significant differences. There are some noteworthy observations. For the out-group, the average for like was 14.9 and trust 12.5. For the in-group, the average for like was 15.1, and trust 13.4. There is an (insignificant) trend towards more trust (lower average) for both groups, possibly because both groups were more typical than the prototype faces. Looking at all the individualized images, we find that 15 out 20 images were ranked higher for trust (paired Wilcoxon test, V = 152.5, N = 20, *p* = 0.0217); however, there is only an insignificant trend for the female images (9 out of 12 images higher for trust, V = 53, N = 12, *p* = 0.0828) and the male images (6 out of 8 images higher for trust, V = 30, N = 8, *p* = 0.1069). One explanation could be that ranking for trust has a smaller span and the prototypes were typically ranked low for trust (6 out of 8 prototypes were lower (“worse”) than the median on trust).

Looking at the prototypes (Table 1: row 1), we note that both female prototypes are well liked (average rank 3.2 and 1.6) but less trusted.

Only eight images reached a significant deviation from the expected ranking of 14. Female prototypes were significantly more liked, as well as the second male prototype. Neutral human prototype one (#5 in row 1) was significantly less liked. Male prototype one, MP1, was significantly less trusted. Male #2 was significantly more trusted, Male #5 was significantly less liked. Additionally, two females might have recognized themselves (#3 and #10 in Table 1: row 3), ranking themselves higher than expected.

We will come back to the correlation between like and trust below.

**Figure 2:**
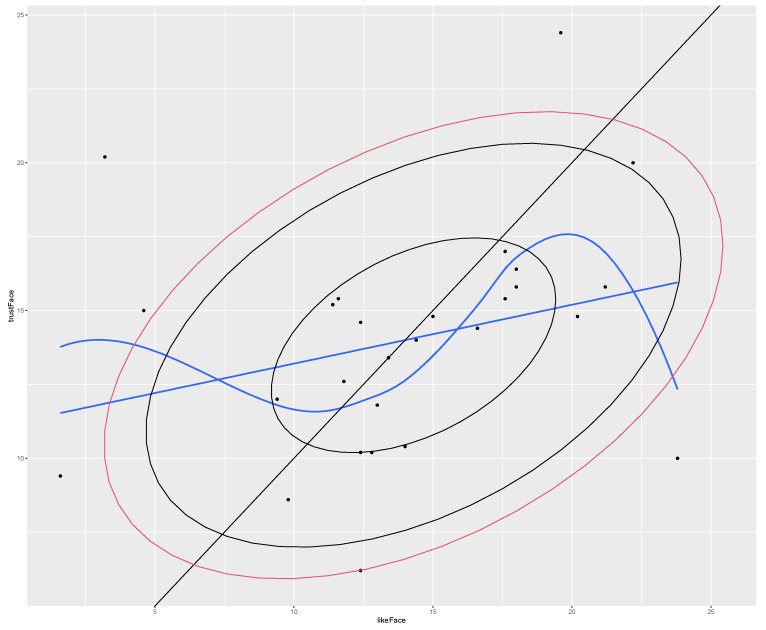
Correlations between average like and trust judgments. The dots mark the average scores of each face.

Figure 2 shows the relation between average scores for each image, for like and trust. The ellipses show the 50%, 90%, and 95% confidence intervals. There are no linear correlations. The black line shows a hypothetical one-to-one relation. The blue line is the linear trend line (not significant). The smooth curve shows that there is some structure, but it is not linear. When the like score is low (more liked), the trust score is up (less liked). In the middle range, we see that as the like ranking gets lower (score is higher), the trust also goes up (more distrusted). Finally, the extremely disliked images become more trusted again, but the last point is an outlier. Within the 90% confidence interval, there is a strong correlation for the more typical faces, i.e., like scores are larger than 5 and smaller than 22, and trust scores are larger than 5 and smaller than 19. This removes all the prototypes, except H2 and Jesus. It also removes Male 5, which is a clear outlier. This gives a significant non-parametric correlation (Spearman’s rho = 0.60, S = 616, *p* = 0.0040) for the more typical faces. This mirrors the prediction of Sofer et al. [14] that typical faces are more positively evaluated for both like and trust. However, the faces in our data are very similar, and we did not aim to create a gradual incline from unattractive to very attractive. Thus, the typical faces may emerge from exposure.

### 3.1. Prototype Effects

Prototypes generally rank higher for beauty because the prototypes cancel out small imperfections and increase symmetry. We observed that five out of seven prototypes ranked higher for beauty than trust, and three of these ranked significantly higher than expected (see Table 2 and Table 3). The individualized images (in Table 3) that ranked high for beauty were from one male and one female individual. 

### 3.2. Individualized Faces Are More Trusted Than Beautiful

Out of 20 individualized faces, 15 ranked higher for trust than beauty. Table 4 shows the faces that rank highest for trust. Typical faces outrank most prototypes. This effect is congruent with a typicality preference, as discussed by Sofer et al. [14].

### 3.3. Self-Recognition

Out of 10 possible, 6 were ranked higher than the average ranking, and 3 were ranked lower. All participants in the like condition ranked themselves higher than their average ranking, but only one was significantly higher by self-ranking. All three males were in the like condition. There are insufficient data to generalize, but it appears that people do react better to faces that are like their own. The differences between faces were small in this experiment, which may indicate that people are highly attuned to facial features. That two people ranked images that included their own facial information significantly higher than the average may indicate that these two people, at some level, recognized themselves. It could also be argued to be a more specific familiarity effect, although the image in the mirror most of us are familiar with is different from a photograph that is not mirrored and thus more like how others see us.

### 3.4. A Model of Face Proportions

A simple model of face proportions was constructed in GeoGebra [22]. The first vertical line is drawn from the midpoint (G) of the highest point of the forehead to the lowest point of the chin (H), constructed such that it follows the nose and passes through the infra-nasal depression (the philtrum) as centrally as possible. The width of the face is estimated by locating the zygomaticus bone, guided by the lower earlobes and the tip of the nose, resulting in the line K-L. The width of the eyes is measured by the line C-D, and symmetry of the eye is measured by comparing C-I to I-D, and the angle of the eye-line to the vertical line. The width of the mouth is similarly constructed by the line E-F, found by locating the oral angles (*anguli oris*) and aligning the line between the upper and lower lip. Mouth symmetry is similarly determined by comparing the distance E-J to J-F, and noting the angle of the mouth line to the vertical line.

**Figure 3:**
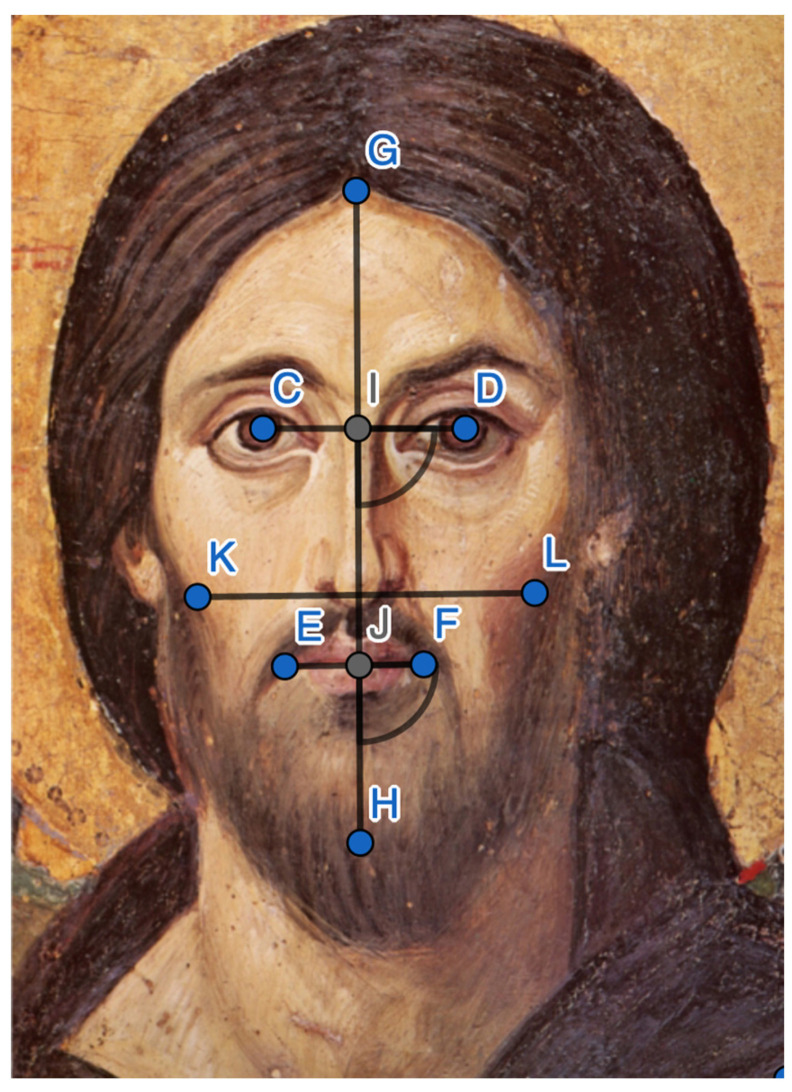
The model for estimating face anatomy, with a background face illustration. The letters mark fixpoints for constructing lines and areas (e.g., a forehead triangle CGD refers 3 points).

Triangles estimate significant proportions of the face. The forehead triangle (CGD) is compared to the upper-face triangle (KLG). The left-side triangle (KGH) is compared to the right-side triangle (GLH). The mid-section (KCDL), including eyes and nose, is compared to the face area (KGLH). All comparisons are scaleless comparisons, i.e., proportions, which avoid the issue of absolute size. All comparisons were made using a common model (Figure 3) adjusted to each individual face image, by the fix points in the face.

We can visualize multi-factorial data on the same scale (here, proportions and percentages) by applying Correspondence Analysis, which presents rows (here, codes for our images) and columns (attributes of the images) projected on the principal component dimensions that best explain the variance of the dataset (confer [23]).

**Figure 4:**
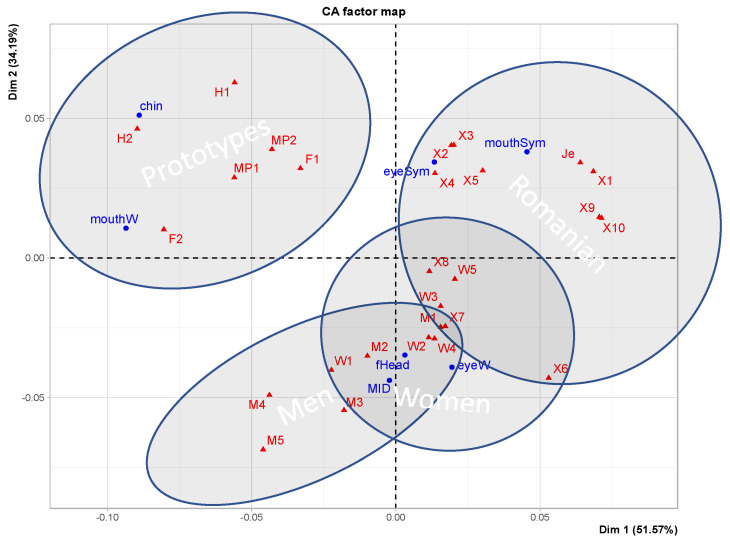
Correspondence Analysis (anatomical features). Red points refer to faces, and blue dots refer to features of faces. (Confer Appendix B).

In Figure 4, we see how anatomical features cluster using a Correspondence Analysis. This demonstrates that the images contain (anatomical) information that makes it possible to differentiate the individual images and detect group differences. Some relevant subgroups are indicated. The prototypes are clearly distinct from the individualized faces. The Romanian students form one recognizable subgroup, with an overlap with women (possibly because there were more women in this subgroup). The Jesus prototype (Je) is central in the Romanian subgroup. Men and women form partly overlapping subgroups, using this set of features. The main dimensions are associated with the more extreme points: along the x-axis, we see mouth width to mouth symmetry, and along the y-axis, we see eye width to eye symmetry. The MID-section is also associated with eyes, and thus gaze. Apparently, the groups separate well, and the two dimensions account for 85.76% of the variance (51.57 + 34.19).

**Figure 5:**
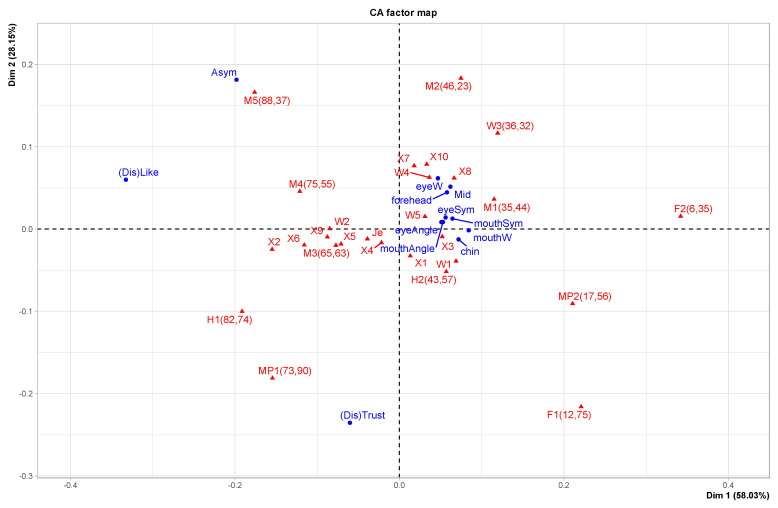
Correspondence Analysis with added average ranking scores. Red points refer to faces, and blue dots refer to features of faces. The numbers for faces far from the expected are the scores for like and trust, as proportions of the maximum score (the lowest rank 27). (Confer Appendix B).

When we add the averaged ranking for each item (Figure 5, see also Table 1), and include the angles of eyes and mouth (as proportions of 180 degrees), as well as add the percent deviance from symmetry (Asym), other patterns emerge in the Correspondence Analysis. We see an association between dislike and asymmetry.

We find all the prototypes represented at the extreme distances from the origin, and most of the males (M#) and some women (W#). The extreme points in Figure 5 have their values on like and trust given (as percent of the maximally low ranking-number (i.e., 27)). Clockwise, we find M2 (at 46% of dislike, and 23% distrust), W3, M1, F2 (female prototype 2), H2 (human prototype), MP2 (male prototype), F1, MP1, H1, M3, M4, and M5. The in-group Romanian students are all near the origin, as are most women, and significantly the Jesus prototype (Je), which is among the closest to origin and part of most of the images. 

**Figure 6:**
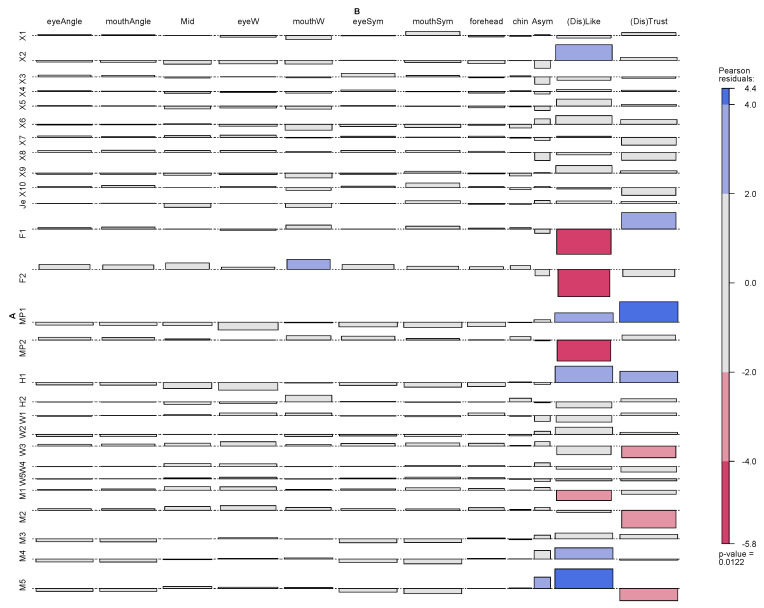
Association table for the correspondences. A are the faces. B are the features of faces.

We see that many anatomical features related to eye and mouth and central face areas contribute to the like side, which may mirror the expressiveness of, and our innate interest in, eyes and mouths. A smile can be noticed at the eyes and at the mouth, and so can a smirk. The graph indicates that there is not a direct one-to-one mapping of facial anatomy to judgments of beauty and trust but rather a holistic impression considering many factors. The association graph (Figure 6) show which features are over and under-represented for which images. The female prototypes are strongly disassociated with dislike (i.e., associated with like). From the other features, we can also see how similar they are (i.e., the bars go in the same direction). The main difference is an association with mouth width, although many other features are also somewhat stronger for F2 (see Figure 6). The M5 has a strong association with asymmetry, which may explain why this image was less liked. We also observe that there sometimes is a dissociation between like and trust, noted by the different direction for dislike and distrust shown in Figure 6.

## 4. Discussion

We confirmed a likeability advantage for female images, which is most notable in the female prototypes. What is surprising is how the first female prototype is liked but not trusted, scoring significantly worse (when tested with an association test) at the bottom of the ranking for trust. This effect for F1 was so noticeable that we took it up in the debriefing discussion with our participants. All participants agreed that F1 was in a sense “beautiful”, but several claimed that there was something off with her eyes, in contrast to her mouth. This could be an example of the “uncanny valley” phenomenon [24,25], i.e., that people are sensitive to incongruent signals, especially in human facial expressions. We also suspect that when the image shows a congruent hint of a smile in both eyes and mouth, it will be more likeable.

Individual images were thus often judged differently in the like and trust conditions. This indicates that we are looking at two dimensions, but they are not entirely independent. We are often told in fairy tales that beauty is also morally good and trustworthy (confer [10]). However, our results tell a different story: trust is not directly related to beauty but is a complicated process of inferring mental states (benevolence) and intentions from fuzzy cues. This is also noted in the fact that very few images are ranked high for trust on average, i.e., there is less consensus on what to look for when judging trustworthiness, and it is not necessarily beauty. As mentioned in the introduction, trust is inspired more by the familiar and the typical face, and this was confirmed in this study. The more extreme faces were generally not trusted, and that applies remarkably to the most beautiful faces. This supports the idea that one feature of trust judgment is detecting what is typical. Different societies may have different defaults for who can be trusted. In a safe society, the bias could be towards typically trusting most people, and thus trusting typical people.

Real trust is a complicated feeling that develops and emerges over time. Being able to quickly and correctly judge whether you can trust a person clearly has an evolutionary value at both the group level and at the pair bonding level, choosing mates, and choosing friends. 

People consistently judge, from a picture, whether a person is trustworthy or not, but they may have different biases. One possibility is that trustworthiness may originate in similarity to known people that participants know and trust, but would there then not be disagreement between in-group and out-group? This study indicates that we are inferring some hidden qualities from observable features in the images, such as perceived benevolence and trust. Even though there was a small but significant correlation between trust and like, trust is clearly not a simple calculation from perceived attractiveness, or simply a judgment of anatomical features. 

There are obviously other variants of the sorting game. We could, for example, ask participants to sort the cards into two mutually exclusive groups: attractive or not attractive, and trustworthy or not trustworthy. This would allow us to use regression models to find out the relations between attractiveness (or trust) and other controlled factors. This would demand more data and more participants but may provide more detailed information. 

The prototypes (row 1 in Table 1) were higher in beauty than in trust (5 of 7), whereas the individualized images were typically higher for trust (15 of 20). This conforms a prototype effect, but prototypes might also feel uncanny and less real, and possibly support the common adage that if something is too good to be true, it is probably not true. Could beauty also be a signal to be careful?

## 5. Conclusions

Our first hypothesis is that trust and like are different; we could not reject the null hypothesis, as the relation between trust and like shows a correlation, but only for the typical faces. We noticed several examples where the average judgments were significantly different. This indicates that like and trust are different but interrelated dimensions. 

Our second hypothesis is that there are differences between male and female images (i.e., rows 2 and 3 in Table 1); we could not reject the null hypothesis. Male individualized images were significantly higher in trust than in like, but there was no consistent difference for female participants. However, it should be noted that seven out of the ten participants were female. 

Our third hypothesis is that participants would react more positively to images that involved themselves. Despite the scarce data, this has some support since two participants had significantly higher ratings for themselves, one in the like condition and one in the trust condition. We expected that self would be a more important factor for trust. Overall, we also observed that many participants did rank themselves higher than the median position. 

Our fourth hypothesis is that participants will react more positively to their in-group compared to an out-group; here, we cannot reject the null hypothesis. The out-group was even rated slightly more trustworthy than the in-group (not significant, but the direction is against the hypothesis).

Symmetry is not the only factor that affects beauty, and our results support a positivity bias, suggested by Sofer et al. [14], for typical faces, and indicate that typical faces can emerge out of exposure within the experiment. It is thus possible for asymmetry to be valued favorably, and by becoming familiar with someone in a positive context, we may become more positively evaluated. Therefore, it is not necessary to correct smaller imperfections to appear more likable, and small asymmetries might not even be noticed, or even positively valued, by others, as noted by Choi: “Favorable facial images can have […] asymmetry, and a natural profile is preferred over a perfect-mirror image. Images with canting less than 3°–4° […] are not recognized as asymmetric in general.” [26] (p. 9).

Lessons learned are that the simple card sorting game can be used to investigate the preferences and biases in human facial recognition. The results presented in this article should be viewed as a demonstration of concept in a small study. The fact that some preferences were significant with only five participants in each group demonstrates both the feasibility of this experimental method and that we are looking at large effects for some of the cards. The method is very time efficient, as the sorting game can be completed within 5 min per participant, and no other special equipment is needed. It is easy to produce multiple stacks of cards, which facilitates running multiple participants in parallel. The cards could be printed and laminated at a cost of less than EUR 2 per card. The stacks of cards do not demand access to electricity or special equipment, which is ideal for field studies. The number of permutations of 27 cards is 27! (~10^28^), so it is remarkable to observe a high consistency between participants in how they sort the cards.

Are prototypes easy on the mind? We have demonstrated a typicality effect and that there is a difference between what is typical and what is prototypical. The two terms might be confused. The prototypical is analogous to the central tendency in a population, and the more data we base the prototypes on, the less uncertainty, just as the standard error of the mean tends to be smaller in a large sample. The typical may have considerable variance, analogous to the uncertainty in data, or standard deviation, in a sample. The typical is common and familiar, and the prototypical is rare and valued. Faces have many dimensions. Thus, when we work with fewer dimensions, we project the data from different angles. However, there ought to be a way of separating what is typical from what is prototypical. The typical has a wide range within normality, and the prototypical is an abstraction. The typical in a sample contains the representative data points. The prototypes in this article are abstractions from a certain bias, e.g., female and male prototypes. The human prototypes had features of both male and female, and may thus be incongruent, which is one explanation why they were valued lower than especially female prototypes. Prototypes may have advantages [27], but we also detect and value what is typical.

We must keep in mind that we were not able to perfectly balance our participants, and all three males were in the group that judged attractiveness, rather than trust. Our sample is small but demonstrates a novel sorting task. However, our findings are in line with previous research, outlined at the start of the article. Further studies may uncover more precise features that bias our judgments. It is, for now, an open question how *accurately* people can judge trustworthiness, especially without any extended interaction with the person. It is highly unlikely that trust can be judged accurately from just an image.

## Figures and Tables

**Table 1:** The images in the first row are: two female, two male, and two neutral prototypes, and the prototype of Jesus. The second row are images from another group of students (out-group). The first five are female, and the last five are male. The last row of images is the in-group of Romanian students: here, images 1, 2, and 5 are underlying male, the others are female. The last line of numbers gives the self-rating for each of the participants, a + represents a higher self-ranking. Two female students gave surprisingly good scores for themselves, one in the like (L) and the other in the trust (T) condition. The expected score was 14. All scores are averaged over five participants, except the self-scores. Boldface marks what is significant from the median, or when trust is better than like.

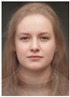	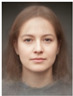	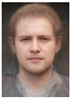	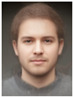	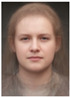	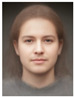	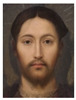			
**3.2**	**1.6**	19.6	**4.6**	**22.2**	11.6	15.0			
20.2	9.4	**24.4**	15.0	20.0	15.4	*14.8*			
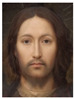	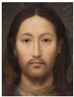	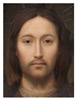	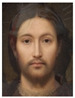	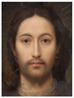	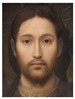	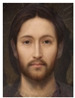	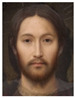	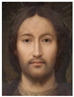	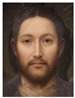
11.4	18.0	9.8	13.0	13.4	9.4	12.4	17.6	20.2	**23.8**
15.2	**15.8**	**8.6**	**11.8**	13.4	12.0	***6.2***	**17.0**	**14.8**	**10.0**
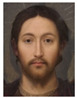	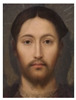	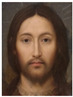	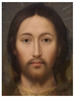	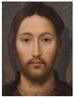	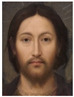	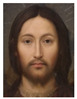	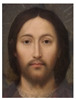	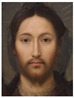	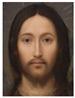
12.4	21.2	11.8	14.4	16.6	18.0	14.0	12.4	17.6	12.8
14.6	**15.8**	12.6	**14.0**	**14.4**	**16.4**	**10.4**	**10.2**	**15.4**	***10.2***
+L 8	L 23	**+L 4**	+L 7	+L 14	T 19	+T 10	+T 11	T 20	**+T 5**

**Table 2:** These five prototypes were rated higher for beauty than for trust. The numbers indicate the average ranking score. The first three are significantly above average (14), the fourth is above average, and the last is significantly below average.

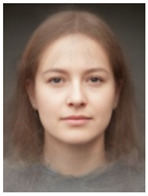	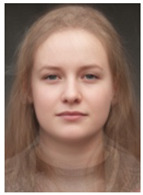	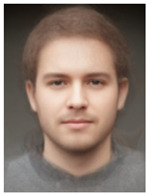	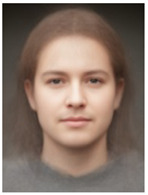	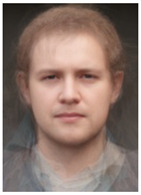
1	2	3	6	26

**Table 3:** These five faces were rated highest for beauty. Numbers 1 and 5 rank high for trust as well.

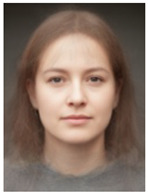	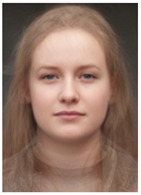	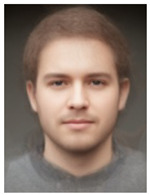	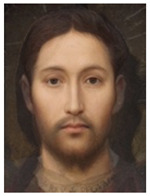	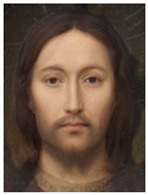
1	2	3	4	5

**Table 4:** The five faces ranking highest for trust. Number 1 is significantly higher for trust than the average. Number 2 is above average for beauty and trust. Number 3 ranks significantly higher for beauty. Number 4 is significantly lower for beauty. Number 5 is significantly higher for self-rated trust.

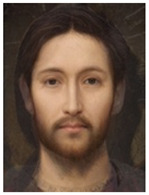	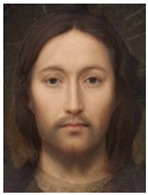	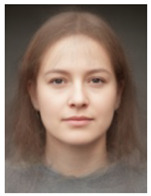	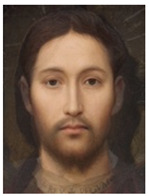	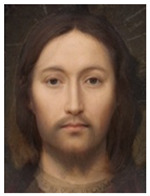
1	2	3	4	5

## Data Availability

All data are available in this publication and/or at request for purposes of research.

## Appendix A

**Table A1:** The ranking for each of the images for each subject (in the like condition (L) and in the trust condition (T)) and the average scores. Bold marks self-ratings.

	L1	L2	L3	L4	L5		T1	T2	T3	T4	T5		T-L
F1	2	1	1	5	7	3.2	3	23	25	27	23	20.2	17.0
F2	1	2	3	1	1	1.6	1	24	6	14	2	9.4	7.8
MP1	16	10	27	20	25	19.6	25	26	27	17	27	24.4	4.8
MP2	5	3	11	2	2	4.6	14	22	13	12	14	15	10.4
H1	20	21	26	21	23	22.2	21	27	26	16	10	20	−2.2
H2	10	11	12	3	22	11.6	5	25	14	15	18	15.4	3.8
X	21	15	13	17	9	15	16	9	6	18	25	14.8	−0.2
W1	4	8	16	10	19	11.4	15	20	22	7	12	15.2	3.8
W2	19	25	20	11	15	18	9	18	7	23	22	15.8	−2.2
W3	9	5	9	6	20	9.8	11	5	5	11	11	8.6	−1.2
W4	3	12	21	18	11	13	7	7	24	1	20	11.8	−1.2
W5	12	27	18	4	6	13.4	12	21	1	9	24	13.4	0.0
M1	7	4	17	15	4	9.4	6	2	12	19	21	12	2.6
M2	6	13	24	16	3	12.4	17	3	2	8	1	6.2	−6.2
M3	11	26	23	23	5	17.6	22	19	3	22	19	17	−0.6
M4	15	9	25	25	27	20.2	10	6	23	26	9	14.8	−5.4
M5	22	22	22	27	26	23.8	2	12	15	13	8	10	−13.8
X1m	**8**	6	7	24	17	12.4	26	1	17	3	26	14.6	2.2
X2m	25	**23**	19	26	13	21.2	18	14	20	21	6	15.8	−5.4
X3	17	17	**4**	13	8	11.8	23	15	16	6	3	12.6	0.8
X4	18	18	5	**7**	24	14.4	24	16	9	5	16	14	−0.4
X5m	13	24	10	22	**14**	16.6	27	8	18	2	17	14.4	−2.2
X6	27	19	14	12	18	18	**19**	4	21	25	13	16.4	−1.6
X7	23	20	8	9	10	14	4	**10**	10	24	4	10.4	0.4
X8	14	7	6	14	21	12.4	8	13	**11**	4	15	10.2	−2.2
X9	26	16	15	19	12	17.6	20	11	19	**20**	7	15.4	−2.2
X10	24	14	2	8	16	12.8	13	17	6	10	**5**	10.2	−2.6

## Appendix B

The data for Correspondence Analysis. All data are scaleless as proportions (%).

Formatted to create a matrix in R for direct further analysis.

Column 1, 2: The eyeAngle and the mouthAngle are proportions of 180 degrees.

Column 3: The proportion for the MIDsection.

Column 4, 5: Eye width and mouth width as proportion of the zygomaticus line.

Column 6, 7: Eye and mouth symmetry. The face’s right side compared to total width of eye/mouth.

Column 8, 9: The proportions for the forehead and chin.

Column 10: The percent absolute deviance from equal proportions of left and right side.

Column 11, 12: The proportion of average rank to maximum (27) for like and trust.

The following gives the code for generating the table in R, for further processing.

It can be accessed by copy from the pdf version, and paste into R.

faceAllC <-matrix(c(

49.42,	49.14,	35.30,	50.00,	29.46,	49.19,	55.49,	20.09,	7.09,	4.40,	45.93,	54.07,
49.57,	49.14,	33.22,	51.56,	32.74,	53.85,	50.35,	19.96,	8.14,	0.84,	78.52,	58.52,
49.58,	49.14,	32.96,	51.56,	32.73,	53.88,	50.37,	20.21,	8.07,	0.81,	43.70,	46.67,
49.32,	48.88,	33.13,	51.56,	32.74,	52.27,	49.33,	20.04,	8.11,	2.90,	53.33,	51.85,
49.67,	49.39,	32.40,	50.67,	31.55,	51.57,	49.82,	20.20,	7.35,	2.12,	61.48,	53.33,
50.09,	50.60,	38.43,	53.27,	29.44,	49.64,	47.94,	21.93,	5.93,	6.62,	66.67,	60.74,
50.65,	49.64,	37.90,	56.54,	34.41,	51.82,	50.30,	22.80,	7.29,	2.62,	51.85,	38.52,
50.85,	51.16,	37.61,	55.56,	35.72,	52.49,	52.26,	22.43,	7.50,	0.50,	45.93,	37.78,
49.65,	49.37,	34.99,	54.28,	30.95,	51.56,	55.95,	22.19,	6.53,	4.53,	65.19,	57.04,
49.65,	50.84,	34.99,	54.28,	30.98,	51.56,	55.99,	22.19,	6.42,	4.53,	47.41,	37.78,
49.59,	50.29,	31.66,	54.75,	30.96,	51.42,	55.52,	21.44,	7.39,	5.42,	55.56,	54.81,
50.10,	50.95,	35.31,	50.72,	39.74,	49.89,	53.70,	22.41,	8.45,	2.17,	11.85,	74.81,
50.59,	50.00,	39.74,	51.21,	43.54,	52.34,	49.45,	21.98,	9.07,	0.96,	5.93,	34.81,
50.51,	50.20,	36.08,	47.69,	38.89,	49.85,	48.36,	20.03,	8.52,	5.65,	72.59,	90.37,
50.31,	50.02,	35.46,	50.93,	38.89,	53.73,	50.05,	20.99,	9.31,	3.52,	17.04,	55.56,
50.32,	50.00,	31.69,	46.76,	38.42,	51.49,	48.19,	19.96,	8.95,	3.64,	82.22,	74.07,
50.20,	50.12,	33.73,	52.66,	44.96,	52.54,	51.27,	22.25,	10.26,	3.83,	42.96,	57.04,
49.54,	50.53,	38.38,	59.51,	39.53,	51.66,	49.93,	25.14,	7.68,	1.63,	42.22,	56.30,
50.16,	49.93,	38.89,	58.27,	35.80,	52.59,	51.38,	23.72,	7.59,	5.77,	66.67,	58.52,
50.16,	50.62,	38.50,	58.90,	36.27,	53.08,	53.63,	23.97,	7.89,	5.80,	36.30,	31.85,
50.15,	50.62,	40.78,	59.52,	36.27,	52.48,	53.58,	23.42,	8.11,	5.80,	48.15,	43.70,
50.53,	50.99,	38.39,	58.28,	36.26,	53.68,	54.89,	23.92,	7.82,	2.97,	49.63,	49.63,
50.23,	50.70,	40.36,	58.89,	36.26,	52.44,	53.89,	23.38,	7.86,	5.21,	34.81,	44.44,
50.23,	50.13,	39.83,	59.52,	38.10,	51.93,	51.30,	23.94,	8.16,	5.20,	45.93,	22.96,
49.80,	49.71,	39.61,	60.88,	38.57,	50.03,	49.96,	24.91,	8.13,	6.12,	65.19,	62.96,
50.12,	50.00,	39.39,	61.03,	40.95,	51.36,	48.61,	25.16,	8.77,	8.43,	74.81,	54.81,
50.12,	50.01,	42.75,	62.05,	40.58,	50.73,	47.74,	24.26,	9.07,	9.66,	88.15,	37.04),

nrow = 12, dimnames = list(c(“eyeAngle”, “mouthAngle”, “Mid”, “eyeW”, “mouthW”, “eyeSym”, “mouthSym”, “forehead”, “chin”, “Asym”, “(Dis)Like”, “(Dis)Trust”), c(“X1”, “X2”, “X3”, “X4”, “X5”, “X6”, “X7”, “X8”, “X9”, “X10”, “Je”, “F1(12,75)”, “F2(6,35)”, “MP1(73,90)”, “MP2(17,56)”, “H1(82,74)”, “H2(43,57)”, “W1”, “W2”, “W3(36,32)”, “W4”, “W5”, “M1(35,44)”, “M2(46,23)”, “M3(65,63)”, “M4(75,55)”, “M5(88,37)”)))
